# Characteristics of Vibrating Fluidization and Transportation for Al_2_O_3_ Powder

**DOI:** 10.3390/ma15062191

**Published:** 2022-03-16

**Authors:** Koichiro Ogata, Tsutomu Harada, Hideo Kawahara, Kazuki Tokumaru, Riho Abe, Eiji Mitani, Koji Mitani

**Affiliations:** 1 Department of Mechanical Engineering, National Institute of Technology, Oita College, 1666 Maki, Oita 870-0152, Japan; tsutomu.3_eleven@icloud.com (T.H.); k-tokumaru@oita-ct.ac.jp (K.T.); r-abe@oita-ct.ac.jp (R.A.); 2 Shipping Department, National Institute of Technology, Oshima College, 1091-1 Komatsu, Suo-Oshima, Oshima 742-2193, Japan; kawahara@oshima-k.ac.jp; 3 SSC Corporation Limited, 26-216 Ushiroji, Tomomachi, Hiroshima 720-0202, Japan; e-mitani@ssc-hvaf.co.jp (E.M.); k-mitani@ssc-hvaf.co.jp (K.M.)

**Keywords:** cohesive powder, vertical vibration, fluidization, dispersion, transportation

## Abstract

This study focused on the vibrating fluidized-bed-type powder feeder used in HVAF thermal spraying equipment. This feeder has been used in thermal spraying equipment and industrial applications. However, particulate materials’ flow mechanism and stable transport characteristics have not been fully understood. This study experimentally investigated the fluidization characteristics, powder dispersion state, and powder transportation characteristics of Al_2_O_3_ particles during vertical vibration fluidization. The material used was Al_2_O_3_ particles of 2.9 μm and 3808 kg/m^3^, classified as the group C particles in the Geldart diagram. As experimental conditions, the fluidized air velocity to the bottom of the powder bed and the vibration intensity in the vertical direction changed. The critical fluidization air velocity was defined to evaluate the generating powder flow by vertical vibrating fluidization. As a result, good fluidization of the powder bed of Al_2_O_3_ was obtained by the vertical vibration, as well as an airflow that was higher than the critical fluidization air velocity. Regarding powder transportation characteristics, it was clarified that the fluidized air velocity at the bottom of the powder dispersion vessel and the pressure difference from the powder dispersion vessel to the transportation part significantly affect the mass flow rate.

## 1. Introduction

In recent years, ceramic materials with particle sizes from micron- to nano-size have been developed and applied to various products such as semiconductors, automobiles, and medical equipment. The reason is that ceramic materials have excellent mechanical, thermal and surface properties such as wear resistance, heat resistance, and corrosion resistance. Thermal spraying is an effective method to utilize the mechanical properties of ceramics. Thermal spraying is a surface treatment method in which metal particles are melted or softened on the surface of a product and then sprayed at high speed and high temperature to form a film and improve the material’s surface modification [1]. This research focuses on the thermal spraying method in which the powder material is sprayed onto the substrate with compressed gas.

The High-Velocity Oxygen Flame (HVOF) method, High-Velocity Air Flame (HVAF) method, cold spray (CS) method, and aerosol deposition (AD) method have been developed as devices for spraying with compressed gas [1,2,3,4,5,6,7,8,9,10,11,12,13,14]. Much research and development have been conducted on HVOF and HVAF spraying equipment [1,2,3,4,5]. HVOF spraying equipment has the powder material sprayed onto the substrate at high speed and high temperature with high-pressure oxygen [1]. Regarding HVAF thermal spraying equipment, the results show that the quality of the film on the substrate is higher than that of HVOF thermal spraying. The reasons for this are that the HVAF sprayed coating had higher mechanical properties such as elastic modulus, higher fracture toughness, and equal or higher abrasion compared to its HVOF-sprayed counterparts [3]. Furthermore, the compressed gas used in HVAF is air, which is cheaper than the oxygen used in HVOF. Therefore, it is expected to reduce operating costs.

Next, in the CS method [6,7,8,9,10,11], the working gas temperature is lower than the material’s melting point or softening temperature. A supersonic flow accelerates the feeding particle material through the Laval nozzle. At that time, the material collides with the substrate at high speed in the solid state to form a film [6]. The cold spray also has some disadvantages compared to other thermal spray techniques. The deposition of coatings using CS is based on the plastic deformation capacity of the particles and the substrate. 

Consequently, CS requires the substrates to have a minimum ductility to produce well-bonded coatings [10]. In addition, the consumption of a large amount of He and N_2_ gas used in the system and expensive equipment are also problems [6]. The AD method is a technology that enables film formation even under conditions close to room temperature [12]. Recent research has achieved high-quality alumina coatings [13,14]. On the other hand, the AD method also has restrictions such as low vacuum conditions for forming a film on the substrate and the high cost of the equipment. 

For this reason, we focused on the HVAF thermal spraying equipment and the research and development conducted in this area [15,16]. The HVAF thermal spraying under development uses kerosene as fuel, which is considered to have a cost advantage over the AD and CS. The equipment includes a swirl-type combustion chamber using sprayed fuel and a fluidized-bed-type powder feeder. However, the flow mechanism and performance of the combustion part and powder feed part have not been clarified. Therefore, in the previous study [17], the relationship between the ignition characteristics, the stability and transition characteristics of the flame, and the equivalence ratio with the combustion temperature and the length of the combustion flame was clarified using a curved impinging spray combustor. On the other hand, the flow mechanism and transport characteristics of the vibrating fluidized-bed-type powder feeder, which is supposed to be used in the development equipment, have not been clarified. 

In this research, we focused on the vibrating fluidized-bed-type powder feeder, which is used for feeding a powder material with compressed gas and can use the functions of the material as it is. For example, acoustic sound fluidization mixes two kinds of powder in the CS method [18]. In the AD method, after the powder filled in the vessel is mixed with gas to form an aerosol by vibration, the aerosol particles are transported by the gas flow generated via the pressure difference between the feeding chamber and the deposition chamber [13,14]. However, micron-sized particles’ flow mechanisms and stable transport characteristics have not been clarified. 

There is much research on vibrating fluidized powder beds [19,20,21,22,23,24]. These studies have investigated the flow pattern [20,24] and flow characteristics [19,20,21,22,23,24] to the particulate materials of micron- and nano-size when the vibrating amplitude, frequency, and fluidized air velocity are changed. Regarding the particle dispersion in the vibrated powder bed, there is also an experimental study of the release of dust from cohesive powder by vibrating fluidization [25,26]. On the other hand, there are no research reports on the transportation characteristics of powder using a vibrating fluidized bed. 

Based on the above background, in this study, we attempted to investigate the operating conditions and elucidate the flow characteristics of a micron-sized ceramic powder dispersed and transported by vibration fluidization. An alumina powder that is classified as being in Group C in the Geldart diagram [27] was used. As for the experimental conditions, the secondary air velocity to the powder feeder was made constant, and the fluidized air velocity to the bottom of the powder bed and the vibration intensity in the vertical direction were changed. This paper reports the results obtained for the fluidization characteristics, powder dispersion state, and powder transportation characteristics of the powder bed during vertical vibration fluidization.

## 2. Experiment

### 2.1. Materials

**Figure 1:**
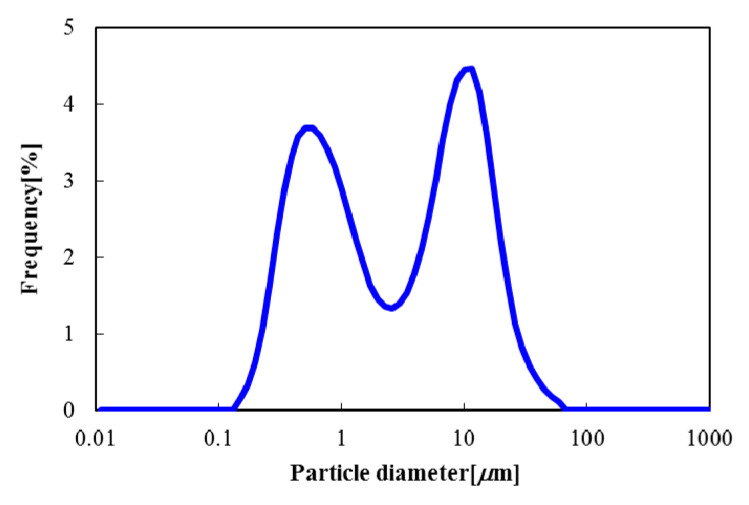
Particle size distribution of Al_2_O_3_ powder used.

This study used Al_2_O_3_ (Sumitomo Chemical Co., Ltd., Tokyo, Japan, AM-27) as the cohesive ceramic powder. The particle size distribution of alumina powder was measured by a laser-diffraction particle size analyzer (HORIBA, LA-950). As for the measurement conditions, the refractive index of the alumina was 1.76, and the refractive index of the distilled water was 1.333. The particles were led to the measurement cell with distilled water. At that time, the particles and distilled water were circulated in the measurement cell while stirring. However, ultrasonic dispersion treatment was not performed to maintain the aggregated particles. The particle size was measured three times at each experimental condition and averaged. Figure 1 shows the result of the particle size distribution of the Al_2_O_3_ used. It can confirm that the primary particle size was less than 1 μm, and the agglomerated particle appeared at around 10 μm. The median particle diameter *D*_50_ was 2.92 μm, and the particle density was 3808 kg/m^3^. Table 1 indicates the measurement result of the flowability of Al_2_O_3_ particles. These particle properties were measured by the Powder Tester (HOSOKAWA MICRON, PT-X). The compressibility and the cohesiveness denoted a high value, and these flowabilities were evaluated as significantly worse. As a result, the flowability index was 26.5, as shown in Table 1. The flowability was assessed as Very Poor based on Carr’s flowability index [28,29,30].

### 2.2. Test Equipment

**Figure 2:**
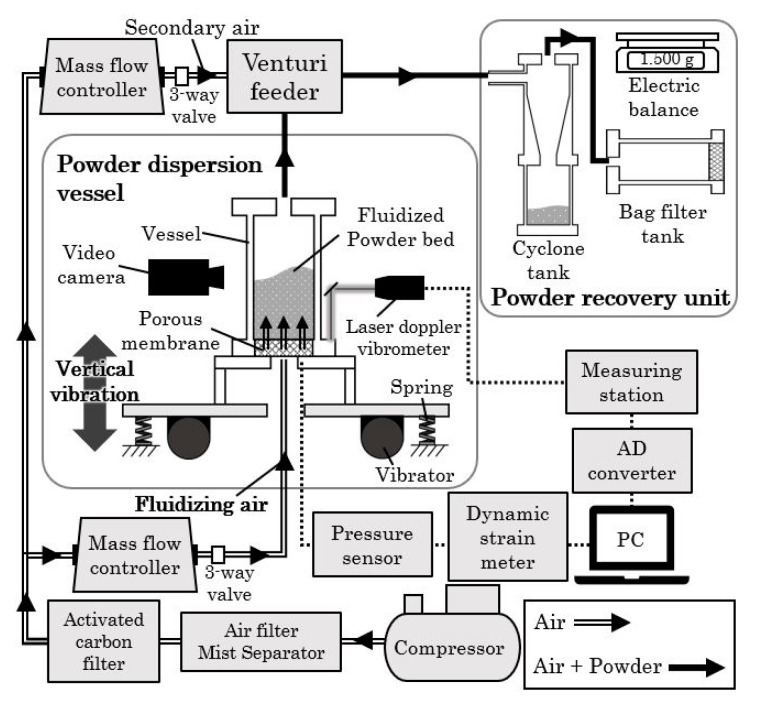
Test equipment for vibrating fluidization and particle transportation.

Figure 2 shows a schematic diagram of the test equipment for the powder transportation system that used vibrating fluidization. The test equipment consisted of a powder dispersion vessel, a venturi-type powder feeder, and a powder recovery unit. A powder dispersion vessel was made using an acrylic cylindrical pipe with an inner diameter of 50 mm, a height of 300 mm, and a thickness of 8 mm. A polyurethane tube with an inner diameter of 4.23 mm was used for the air supply and the powder feeder.

A porous membrane of 6 mm thickness was installed at the air supply part of the bottom of the powder dispersion vessel to provide uniform air for fluidization. Furthermore, two vibration motors were connected at the bottom of the dispersion vessel with an iron plate and the vibration spring to give vertical vibration to the powder dispersion vessel. The vibration motor (URAS TECHNO, SEE-0.5) had a weight inside and could be adjusted from 0% to 100%. In this experiment, the motor weights were 54%, 71%, 86%, and 100%.

A Venturi-type feeder was installed above the powder dispersion vessel to assist in transporting powder from the powder dispersion vessel to the receiving tank. The fluidized air from the bottom of the powder bed into the powder dispersion vessel and the secondary air from the upstream side of the Venturi feeder were exhausted through the filter at the end of the powder-receiving tank. Here, the internal shape of the Venturi-type powder feeder was 7 mm in diameter on the upstream side, e.g., the secondary air introduction section, the flow path was tapered toward the downstream direction, and the throttle section was 4 mm in diameter. Further, the flow path was restored by taper from the throttle part, and the diameter on the downstream side was 7 mm, which was the same as the upstream side. The powder-receiving unit consisted of a cyclone and a receiving tank with a bug filter.

### 2.3. Experimental Conditions

In this study, the vibrating fluidization experiments and the powder transportation experiments were carried out. The alumina powder used in the fluidization and the powder transportation experiments had a median particle diameter of 2.92 μm, a particle density of 3808 kg/m^3^, and an initial filling amount of 100 g in the dispersion vessel. Table 2 shows the experimental conditions of the vibrating fluidization for Al_2_O_3_ particles, where *f* is the frequency, *u_b_* is the fluidizing air velocity at the bottom of the powder dispersion vessel, *u_t_* is the secondary air velocity on the upstream side of the Venturi-type feeder, and *Λ* is the vibration strength.

Table 3 shows the conditions of the powder transportation experiment using vibrating fluidization. Although the parameters were the same as those in the fluidization experiment, this experiment was conducted with fluidized air velocity and vibration intensity, which enabled powder transportation. 

The vibration strength *Λ* was calculated from Equation (1) after measuring the vibration amplitude *A*. Here, *g* is the gravitational acceleration.

(1) $$\Lambda =\frac{A{\left(2\pi f\right)}^{2}}{g}$$

**Figure 3:**
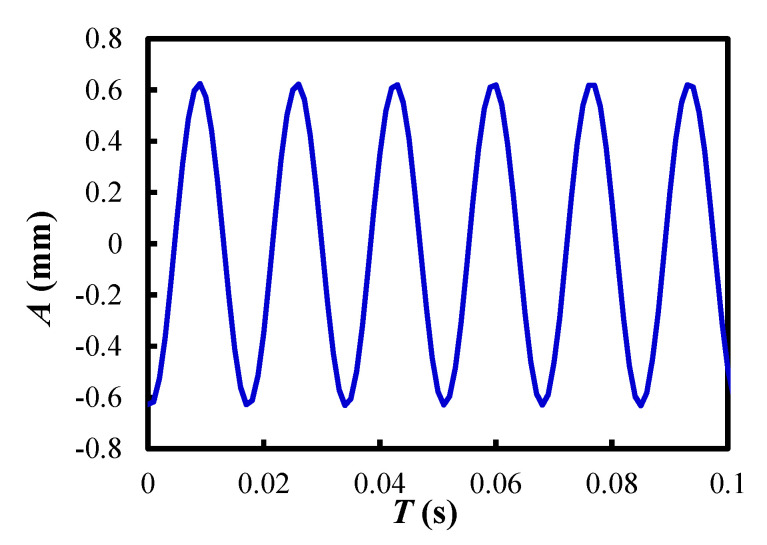
Relationship between vibration amplitude and elapsed time up to 0.1 s, where *Λ* = 9.23.

Regarding the amplitude, the maximum value of the vibration amplitude, measured at intervals of 10 s using a laser Doppler vibrometer, was used. The amplitude *A* of vertical vibration at 60 Hz was obtained using a laser Doppler vibrometer, as shown in Figure 3. The measurement point was chosen for the lower flange of the dispersion vessel to obtain accurate data of vertical vibration to the particles inside the dispersion vessel. The recorded result displayed that the maximum amplitude reached 0.637 mm in the case of the motor weight of 100%, and the vibration strength was 9.23. 

Table 4 indicates the vibration strength when changing the weight of the vibration motor in the present experiment. The vibration strength could be set from 5.0 to 9.23.

### 2.4. Experimental Method

#### 2.4.1. Fluidization Experiment

The fluidization characteristics of Al_2_O_3_ particles were investigated via a de-fluidization experiment, which is less susceptible to inter-particle forces [20]. In the experiment, after filling the dispersion vessel with Al_2_O_3_ particles, vertical vibration was applied to simultaneously supply fluidized air and secondary air at the bottom of the powder bed to fluidize the powder bed for 5 min. Next, the fluidizing air velocity gradually reduced from the set velocity, and the pressure at the bottom of the dispersion vessel was also measured at each air velocity. The pressure drop Δ*P* inside the powder bed was calculated using the following equation.

(2) $$\Delta P=p_{d}-p_{a}$$

Here, *p_d_* is the pressure when the powder is filled inside the dispersion vessel, and *p_a_* is the air pressure without the powder in the dispersion vessel.

In this study, the fluidization characteristics of Al_2_O_3_ particles were evaluated from the pressure drop and the fluidization state of the powder bed when the fluidization air velocity of the dispersion vessel was reduced at regular intervals. A digital video camera was used to visualize the fluidized state.

#### 2.4.2. Powder Transportation Experiment

Here, the procedure for powder transportation experiments using the vibration fluidization operation is described. Al_2_O_3_ particles were poured naturally into a cylindrical dispersion vessel. The dispersion vessel was vibrated vertically by turning on the vibration motors attached to an iron plate at the bottom of the dispersion vessel. At the same time, the three-way valve opened to provide the secondary air, and then the valve of the fluidized air was also introduced to supply air toward the porous membrane at the bottom of the dispersion vessel. The Al_2_O_3_ particles entering the dispersion vessel could be dispersed and supplied based on this operation.

The mass of the particles was collected in the cyclone and the tank with a bag filter. The transportation mass of the powder was measured an electronic balance (Shimadzu, UX-2200H) every minute after starting the vibrating fluidization. The experiment was repeated five times, and the mass flow rate *G_s_* was calculated by the relationship between the transportation mass of the powder *M_p_* and the elapsed time *T*.

Furthermore, in this experiment, the particle size inside a dispersion vessel after the powder transportation experiment was measured to confirm the particle agglomeration before and after vibrating fluidization. Samples were taken from the dispersion vessel, and the particle size was measured with a laser-diffraction-type particle size analyzer as in Section 2.1.

## 3. Results

### 3.1. Fluidization Characteristics

Generally, the group C particles in the Geldart diagram have poor flowability. Therefore, it was necessary to confirm the fluidization characteristics of the alumina powder used in this study. For this reason, this section describes the fluidization characteristics of alumina powder when there was no vibration and when vibration was applied. Here, the de-fluidization experiment was conducted as described in Section 2.4.1. The pressure drop was measured, and the fluidized state of the powder bed inside a dispersion vessel was also visualized during the fluidization experiment.

**Figure 4:**
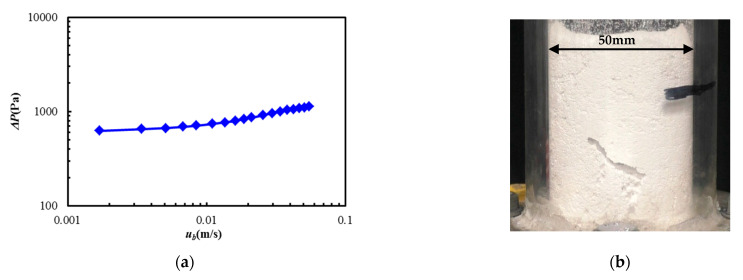
Flow characteristics of the powder bed of Al_2_O_3_ due to the fluidization without vibration. (**a**) Relation of the pressure drop and the fluidizing velocity. (**b**) Visualization of the powder bed (*u_b_* = 0.055 m/s).

Figure 4a shows the relationship between the powder bed Δ*P* and the fluidizing air velocity at the bottom of the dispersion vessel *u_b_* in the case of no vibration. It was found that the pressure drop gradually decreased with the decreasing of the fluidized air velocity. In general, the group A and B particles in the Geldart classification have easy fluidization characteristics. The pressure drop curve showed that the pressure drop took a constant value under the high-velocity condition, and the pressure decreased linearly with the decreasing of the fluidization velocity. At that time, the minimum fluidization velocity could be defined from the pressure drop curve. However, the pressure drop curve of the alumina powder, which belongs to the group C particle, showed different tendencies. 

To confirm this, we visualized the fluidized state of alumina powder without vibration. Figure 4b shows a snapshot of the flow state of the powder bed under the condition of fluidizing air velocity *u_b_* = 0.055 m/s. It can be observed that fluidization of the powder bed did not occur, and that fixed thin channels were formed inside the bed. This was because the adhesive force on the particles had a strong effect, and the entire powder bed could not be fluidized only with fluidizing air. As a result, as shown in Figure 4b, the fluidized state was confirmed over the applied fluidizing air velocity range. The result indicated that the fluidization of the alumina powder used in the present study was difficult in non-vibration conditions. Therefore, it was concluded that a vibration fluidization operation is required to easily fluidize and disperse the alumina powder for the group C particles in the Geldart diagram.

**Figure 5:**
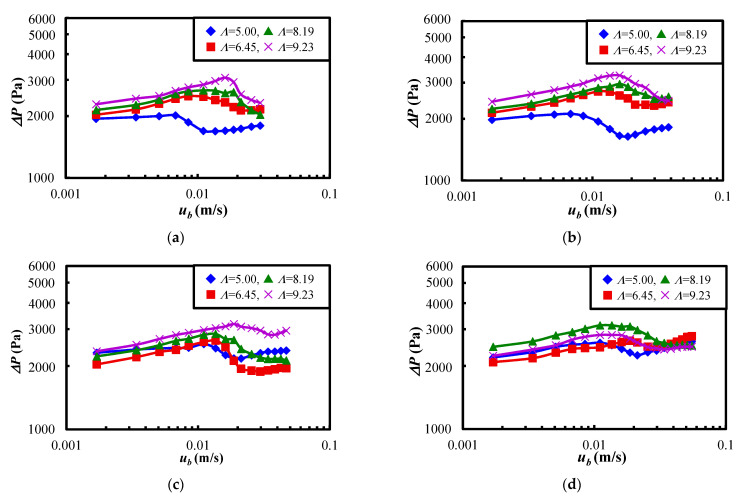
Relationship between the pressure drop of the powder bed of Al_2_O_3_ and the fluidizing air velocity at the bottom of vessel when the fluidization velocity at the start of measurement and the vibration strength were changed. (**a**) *u_s_* = 0.030 m/s. (**b**) *u_s_* = 0.038 m/s. (**c**) *u_s_* = 0.047 m/s. (**d**) *u_s_* = 0.055 m/s.

Next, the results of vertical vibrating fluidization are described. Figure 5 shows the relationship between the pressure drop of the powder bed ∆*P* and the fluidizing air velocity at the bottom of the powder dispersion vessel *u_b_* when the vertical vibration was applied. In this experiment, the powder bed was vibrated vertically and fluidized for 5 min at a fluidized air velocity of *u_b_* = 0.055 m/s. Afterwards, four kinds of measurement were con-ducted with the air velocity us set from 0.030 m/s to 0.055 m/s, as shown in Figure 5a–d, because of the examination of the effect of the starting air velocity on the vertical vibrating fluidization in this study. The pressure drop of the powder bed of Al_2_O_3_ was measured at each fluidization velocity in the case of the same vibration strength. Here, the vibration strength *Λ* was from 5 to 9.23, as shown in Table 4. 

As shown in Figure 5a, the pressure drop of *Λ* = 5.0 gradually decreased up to the air velocity of *u_b_* = 0.01 m/s, and the pressure increased rapidly with the decreasing of the velocity, and then the pressure drop took a maximum value close to the velocity of *u_b_* = 0.007 m/s. Regarding the other vibration strength conditions of Figure 5a, the pressure drop increased with the reducing of the fluidization velocity and reached a maximum value at specific fluidizing air velocities for each vibration strength. This was because the powder bed of Al_2_O_3_ in the vessel transferred from the fluidization state to the expansion state as the fluidizing air velocity decreased. Additionally, the pressure drop after the maximum pressure decreased with the decreasing of the fluidizing air velocity at all the vibration strength conditions, and then the state of the powder bed became close to the fixed bed. The pressure drop curve seemed to have some variability when the starting velocity was changed, as shown in Figure 5b–d. However, the pressure drop curve was almost similar to Figure 5a. Therefore, the same powder behavior appeared in this study’s vertical vibration experiments. 

**Figure 6:**
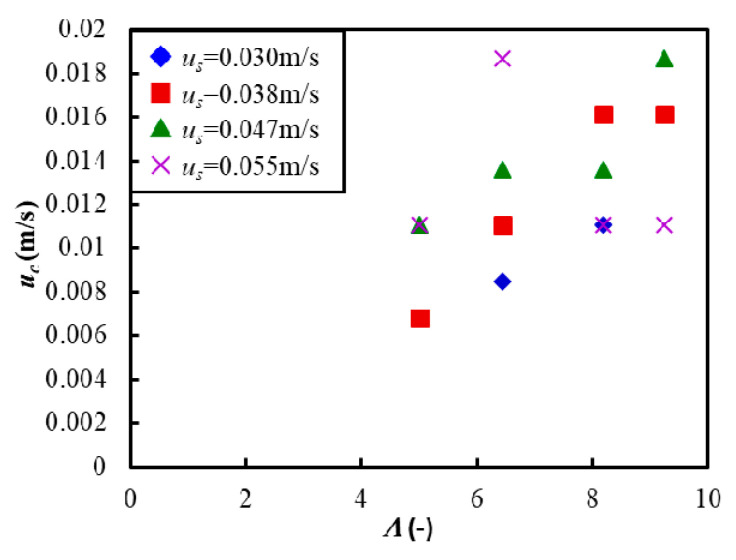
Critical fluidization air velocity during the vibrating fluidization against the vibration strength where the fluidization velocity at the start of measurement *u_s_* = 0.03~0.055 m/s.

In addition, the critical fluidization velocity *u_c_* was defined to discuss the critical conditions for vibrating fluidization of Al_2_O_3_ powder. Here, the critical velocity was estimated as the fluidizing air velocity at the maximum pressure in Figure 5a–d. Figure 6 shows the relationship between the critical fluidization air velocity *u_c_* and the vibration strength *Λ*. Here, the fluidization velocity was changed at the start of measurement *u_s_*. The figure shows that the critical fluidization air velocity increased as the vibration strength increased, although there was some variability. The reason was presumed to be that the powder bed was strongly compressed by vertical vibration when the vibration intensity increased. On the other hand, there was no significant effect of the fluidized velocity at the start of measurement in the experimental conditions of this study.

**Figure 7:**
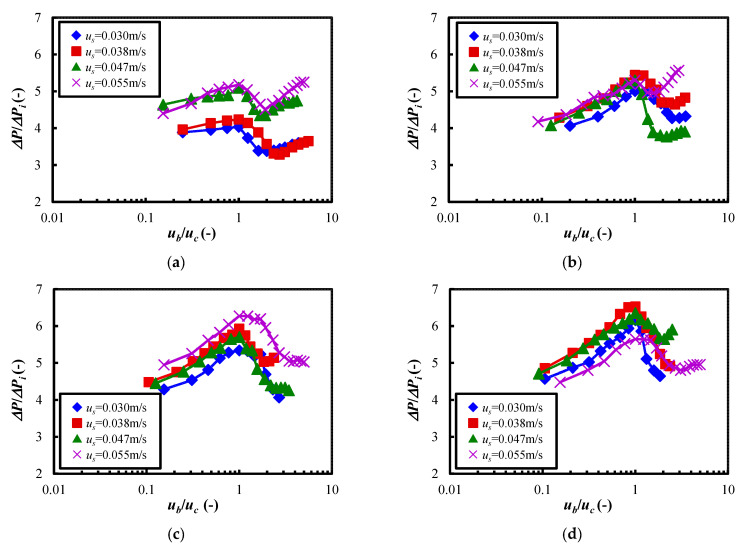
Relationship between the normalized pressure drop and the normalized fluidizing velocity at each vibration strength when the fluidization velocity at the start of measurement and the vibration strength were changed. (**a**) *Λ* = 5.0. (**b**) *Λ* = 6.45. (**c**) *Λ* = 8.19. (**d**) *Λ* = 9.23.

Figure 7 shows the relationship between the normalized pressure drop Δ*P*/Δ*P_i_* and the normalized fluidizing air velocity *u_b_*/*u_c_* when the air velocity at the start of measurement *u_s_* was changed. Here, *u_c_* is the above-mentioned critical fluidization air velocity. The pressure Δ*P_i_*, defined as the gravity at the initial packing of the powder bed, is divided by the cross-sectional area, as expressed by Equation (3). In the equation, *M_pi_* is the initial filling mass of the powder, *A* is the cross-sectional area of the dispersion vessel, and *D* is the inner diameter of the particle dispersion vessel.

(3) $$\Delta P_{i}=\frac{M_{pi}g}{A}=\frac{4M_{pi}g}{\pi D^{2}}$$

The pressure drop acting on the powder bed Δ*P* is the adhesive force of the Al_2_O_3_ powder used in this study and the compressive force due to vibration. The maximum value of Δ*P* is the required pressure to transfer the powder flow from the expanded state to the fluidization state. Therefore, Δ*P*ΔPi can be interpreted as an index showing the conditions for generating powder flow due to the vibrating fluidization used in this study.

In Figure 7a–d, it can be seen that Δ*P*Δ*P_i_* decreased sharply as *u_b_*/*u_c_* exceeded 1.0, regardless of the fluidized air velocity at the start of measurement. This trend showed that the inter-particle and the particle-wall surface forces were reduced by the vertical vibration and the supplied air velocity to the powder bed. At this time, it was observed that the flow pattern of the powder bed was transferred from the expansion bed to the fluidization bed. Furthermore, Δ*P*Δ*P_i_* tended to reach a constant state or increase when *u_b_*/*u_c_* increased. Here, the finding that Δ*P*Δ*P_i_* was close to constant shows the expanded and fluidized state of the powder bed as seen in the group A and B particles in the Geldart diagram. On the other hand, as for the increase in Δ*P*Δ*P_i_*, it could be speculated that the reaggregation of particles may have occurred in the powder bed due to vibrating fluidization. From the above results, it is clear that to obtain the satisfactory fluidization state of the Al_2_O_3_ powder used in this study, it is necessary to apply vertical vibration and, at the same time, supply an airflow higher than the critical fluidization air velocity.

**Figure 8:**
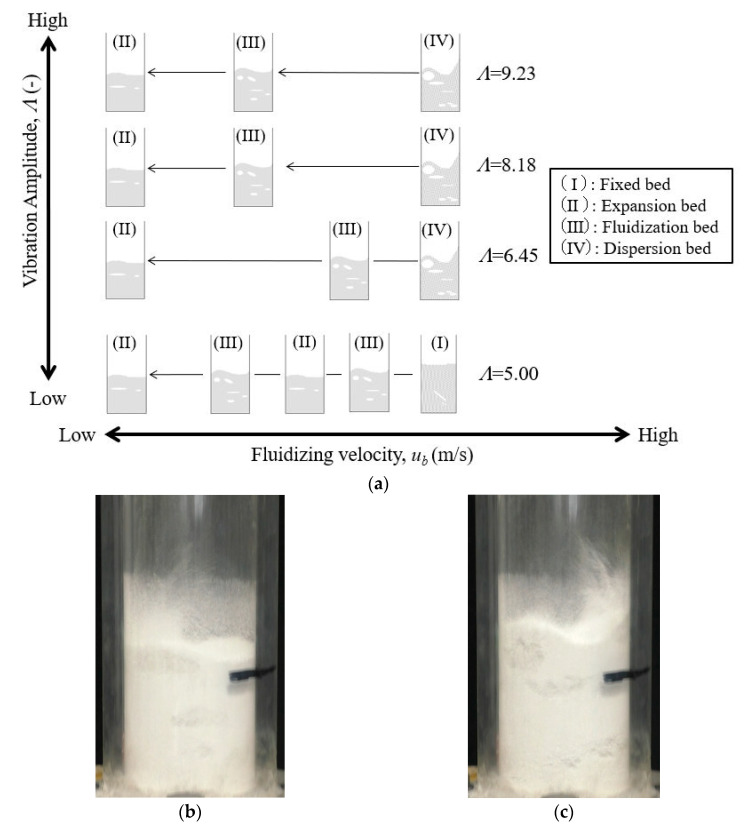
Classification of flow pattern and snapshots of Al_2_O_3_ by vertical vibrating fluidization. (**a**) Classification of flow pattern. (**b**) Fluidization bed (Region III). (**c**) Dispersion bed (Region IV).

Next, the flow state of the powder bed during vertical vibration fluidization obtained from the experiment is summarized. Figure 8a shows the typical flow pattern of the transition state of the powder bed caused by the decrease in the fluidized air velocity *u_b_* at the bottom of the dispersion vessel as a schematic diagram for each vibration strength. The evaluation criteria for the flow state in the figure were as follows.

(I) Fixed bed: The powder bed was close to a stationary state, and agitation or expansion due to the vibration and fluidization of air was not observed.
(II) Expansion bed: Expansion of the powder bed was confirmed by vibrating fluidization. There were no bubbles in the powder bed.
(III) Fluidized bed: The powder bed was fluidized, and air bubbles were also observed, as shown in Figure 8b.
(IV) Dispersion bed: The powder bed was intensely fluidized, and the powder was dispersed toward the upside of the powder dispersion vessel, as shown in Figure 8c.

Complete fluidization could not obtain all the investigated experimental conditions in this study. Figure 8a confirmed that the powder bed expanded and fluidized under the condition of the minimum vibration intensity (*Λ* = 5.0) in this experiment, but a fixed bed was generated again when a high fluidized air velocity was applied. This behavior occurred when the vibration strength given to the powder bed was low. In this condition, the powder flow became unstable. As a result, the stable fluidization and dispersion operation of the Al_2_O_3_ powder was complex in the condition. On the other hand, it was clarified that relatively good fluidization and dispersion could be obtained when the vibration intensity *Λ* ≥ 6.45 and the fluidized air velocity was high. Based on this result, the powder transportation experiment was conducted by means of the vibrating fluidization of Al_2_O_3_ powder under the condition of a vibration intensity *Λ* ≥ 6.45.

### 3.2. Powder Transportation Characteristics

**Figure 9:**
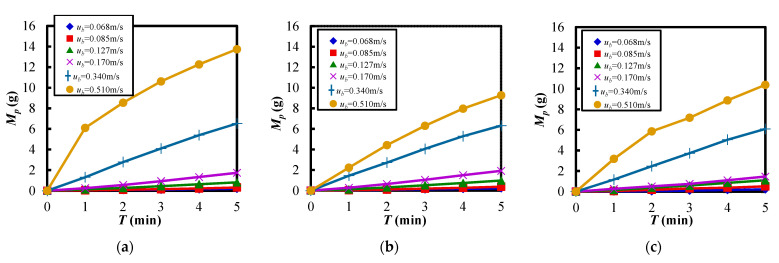
Transported mass of the powder against the elapsed time in the case of different fluidizing air velocities at each vibration strength. (**a**) *Λ* = 6.45. (**b**) *Λ* = 8.19. (**c**) *Λ* = 9.23.

Figure 9 shows the relationship between the transported mass of the powder *M_p_* and the elapsed time *T* when the fluidizing air velocity *u_b_* was changed. Figure 9a–c show the transported mass of the powder in the case of the different vibration strengths. Here, the fluidized air velocity was varied within the range of Table 3. From these figures, it can be seen that the transportation amount increased with the increasing of the fluidizing air velocity at the bottom of the powder bed. Furthermore, except for the conditions of *Λ* = 6.45 and *u_b_* = 0.510 m/s in Figure 9a, the amount of powder transportation increased almost linearly with the elapsed time. It was confirmed that the stable transportation of Al_2_O_3_ powder was realized using the vertical vibration and fluidization operation, as shown in Figure 9a–c. On the other hand, in the case of *u_b_* = 0.510 m/s in Figure 9a, the powder transportation amount increased significantly after the start of the experiment. 

**Figure 10:**
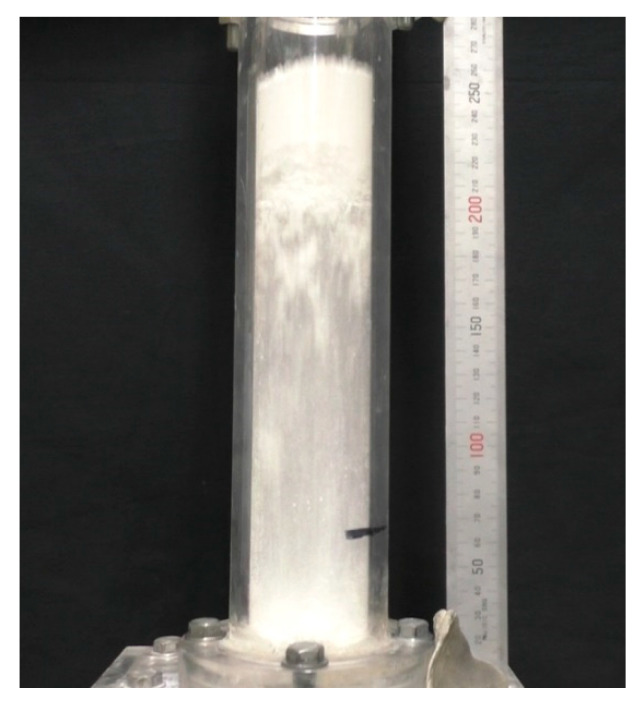
Ascending phenomenon of the powder bed during the vibrating fluidization in the case of *Λ* = 6.45, *u_b_* = 0.510 m/s.

Figure 10 shows the snapshot of the powder flow in the dispersion vessel at this time. From the figure, it can be confirmed that the entire powder bed rose to the upside part of the dispersion vessel after the fluidized air was supplied. Then, the powder bed collided with the top of the powder dispersion vessel, some powder was supplied to the collector, and the remaining powder fell to the bottom of the dispersion vessel to be dispersed and supplied. From this, it was found that the quantitative supply for this study could not be realized under these conditions. Furthermore, it was suggested that the powder bed might become unstable if a high fluidized air velocity is applied under low vibration intensity. Therefore, it was confirmed that care must be taken when setting the conditions for stable transportation of the cohesive powder, such as the group C powder in the Geldart diagram.

**Figure 11:**
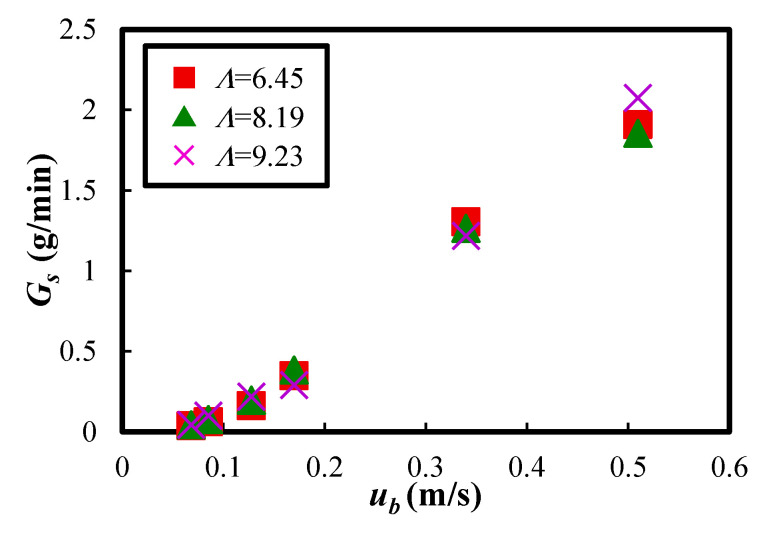
Relationship between the mass flow rate of the powder and the fluidizing air velocity at the bottom of the dispersion vessel when the vibration strength was changed.

Figure 11 shows the results of the mass flow rate of the Al_2_O_3_ powder *G_s_* when the fluidized air velocity *u_b_* and vibration strength *Λ* were changed. Here, the mass flow rate defined the increment of the powder transport amount per minute and was calculated as the average value for 5 min. However, when *Λ* = 6.45 and *u_b_* = 0.510 m/s, the powder transport amount increased suddenly, as described above in Figure 9a, so the mass flow rate was calculated as an average of the data over 4 min, excluding the data for the first 1 min. The result indicated that the mass flow rate of the powder increased as the fluidizing air velocity increased when the vibration strength was constant. Furthermore, the mass flow rate of the powder took the almost same value when the fluidizing air velocity was constant, and the vibration strength was varied. This means that the vibration strength did not significantly affect the change of the mass flow rate in the experimental condition of this study. On the other hand, it was found that the fluidizing air velocity significantly affected the stable powder transportation of the cohesive Al_2_O_3_ powder.

**Figure 12:**
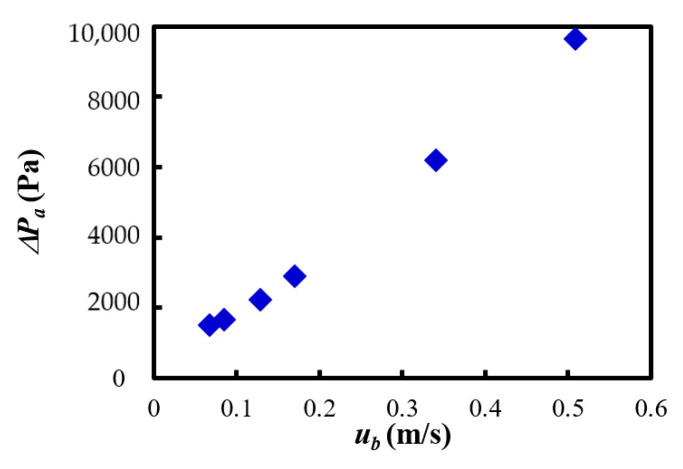
Pressure difference between the exit of venturi feeder and the inlet of cyclone against fluidizing air velocity.

Figure 12 indicates the relationship between the fluidizing air velocity *u_b_* and the pressure difference Δ*P_a_* inside the powder transportation device. The pressure is the differential pressure between the exit of the venturi feeder and the inlet of the cyclone in the case of no filling powder in Figure 2. The pressure difference tended to increase with the increasing of the fluidizing air velocity. In addition, the relationship between the mass flow rate *G_s_* and the pressure difference Δ*P_a_* was examined. 

**Figure 13:**
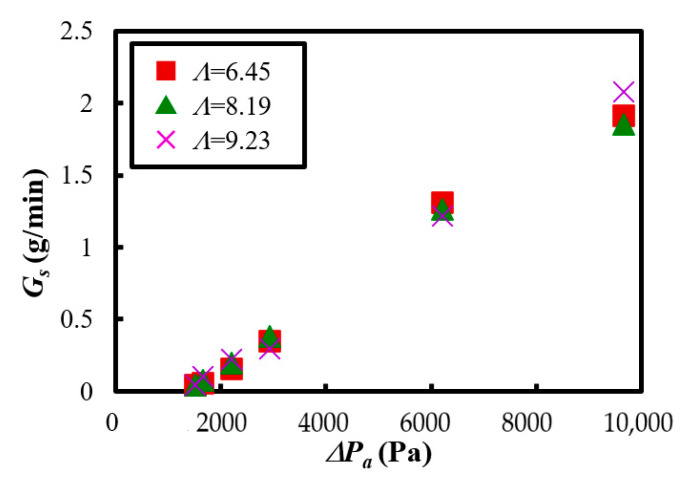
Relationship between the mass flow rate and the pressure difference in the case of the vibration strength *Λ* = 6.45~9.23.

Figure 13 shows the result of the mass flow rate and the pressure difference when the vibration strength *Λ* was changed. It was revealed that the mass flow rate was increased linearly with the increasing of the pressure difference between the feeder and the cyclone. Therefore, it was necessary to increase the pressure difference between the feeder and the cyclone to increase the mass flow rate of the Al_2_O_3_ powder. Furthermore, this device anticipated the operating conditions of the mass flow rate of 1 g/min or more. Regarding this requirement, it was necessary to maintain a pressure difference over 5000 Pa (Figure 13). Figure 11, Figure 12 and Figure 13 show that the powder’s mass flow rate was strongly related to the fluidizing velocity and the pressure. In future work, we will try to introduce a mathematical model for the powder transportation of fluidized-type powder feeders.

### 3.3. Dispersion Characteristics

**Figure 14:**
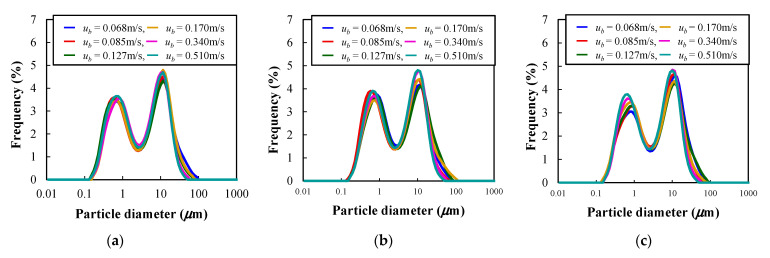
Particle size distribution after vertical vibrating fluidization at each vibration strength and fluidization velocity. (**a**) *Λ* = 6.4. (**b**) *Λ* = 8.19. (**c**) *Λ* = 9.23.

Figure 14a–c indicate the particle size distribution in the powder dispersion vessel after the vertical vibration fluidization when the vibration strength and the fluidization velocity were changed. From all the results, it was found that the distribution had two identical peaks that were the same as the original particle diameter of Figure 1. The trend was almost similar to that observed before vibrating fluidization in that the primary particle size was also less than 1 μm, and the agglomerated particle appeared at around 10 μm. However, the distinction of the particle diameter in the case of changing experimental conditions, such as vibration strength and the fluidizing velocity, was not clear. Therefore, the median particle diameter was plotted against the fluidization velocity at each vibration strength. 

**Figure 15:**
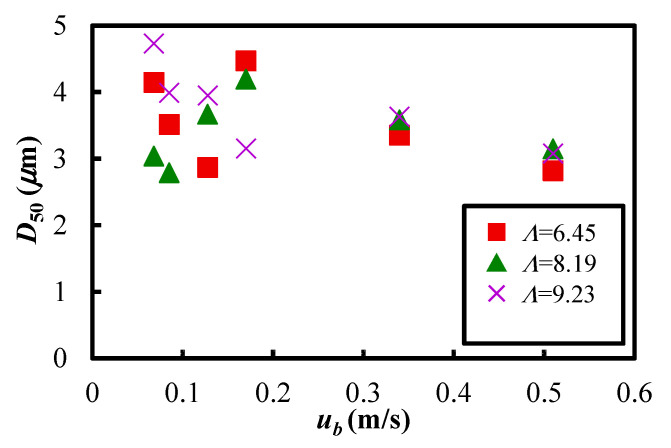
Relationship between the median particle diameter after the vibrating fluidization in the powder dispersion vessel and the fluidizing air velocity (*Λ* = 6.45~9.23).

After the vibrating fluidization experiment, the particles were collected from the powder dispersion vessel and the particle diameter was measured at each experimental condition. Figure 15 shows the relationship between the median particle diameter *D*_50_ of Al_2_O_3_ and the fluidized air velocity *u_b_*. Although there are some variations in the figure, the median particle diameter was about 3 to 5 μm. As described in Section 2.1, the median particle diameter of Al_2_O_3_ powder used in this study was 2.92 μm. This means that the particle diameters before and after vibrating fluidization did not change significantly. Therefore, it is suspected that the Al_2_O_3_ powder was well dispersed and transported to the receiving tank. However, the variations of the particle diameter became prominent in the case of the lower fluidizing air velocity. It is possible that the fluidization of the powder bed was insufficient when the fluidization air velocity was low. In addition, there was no correlation between median particle diameter and vibration strength in the range of experimental conditions in this study.

## 4. Conclusions

This study evaluated the fluidization characteristics, dispersion characteristics, and transportation characteristics of Al_2_O_3_ powder using vertical vibration fluidization. The results obtained are shown below.

(1) It was confirmed that fluidization was difficult for the Al_2_O_3_ used in this study based on the measurement of the pressure drop and the visualization of the powder bed. Therefore, vibration was necessary for easy fluidization and dispersion in the case of the material used in this system.
(2) In order to evaluate the powder flow of Al_2_O_3_ generated by vertical vibration fluidization, the critical fluidization air velocity was defined. As a result, to start the fluidization of the powder bed of Al_2_O_3_, it was necessary to supply an airflow higher than the critical fluidization air velocity together with the vertical vibration. Therefore, it was important to understand the critical fluidization velocity of the materials used. In addition, it was clarified that favourable fluidization and dispersion in the powder bed could be obtained under the conditions of vibration intensity *Λ* ≥ 6.45 and high fluidization air velocity within the range of this experiment.
(3) The powder transport experiment confirmed that the stable transportation of Al_2_O_3_ was realized by using vertical vibration and fluidization operations. Furthermore, it was clarified that the fluidized air velocity at the bottom of the powder dispersion vessel and the pressure difference from the powder dispersion vessel to the transportation part significantly affected the mass flow rate. On the other hand, it was found that when a high fluidized air velocity was applied under the condition of low vibration strength, the powder bed could become unstable, and thus, care must be taken when setting the operating conditions.
(4) From the particle size distribution analysis results in the particle dispersion vessel, it was found that the median particle diameter underwent no significant change before and after vibrating fluidization in the experimental conditions of this study. The results show that favourable powder flow could be obtained in the present experimental system.

## Figures and Tables

**Table 1:** Measurement results of Carr’s flowability index.

Powder Characteristics	Al_2_O_3_	
	Measured Value	Points
Angle of Repose (deg)	46	14.5
Compressibility (%)	51.8	0
Angle of Spatula (deg)	66.9	12
Cohesiveness (%)	84.1	0
Flowability Index (-)	26.5	
Flowability	Very Poor	

**Table 2:** Experimental conditions of vibrating fluidization.

Frequency *f* (Hz)	Fluidizing Air Velocity *u_b_* (m/s)	Secondary Air Velocity *u_t_* (m/s)	Vibration Strength *Λ* (–)
60	0.0017~0.055	3.03	0~9.23

**Table 3:** Experimental conditions for powder transportation.

Frequency *f* (Hz)	Fluidizing Air Velocity *u_b_* (m/s)	Secondary Air Velocity *u_t_* (m/s)	Vibration Strength *Λ* (–)
60	0.068~0.510	3.03	6.45~9.23

**Table 4:** Calculation results of vibration strength.

Motor Weight (%)	54	71	86	100
Amplitude, *A* (mm)	0.345	0.445	0.565	0.637
Vibration strength, *Λ* (–)	5.0	6.45	8.19	9.23

## Data Availability

Not applicable.
